# Simulation Research on Low-Frequency Magnetic Noise in Fe-Based Nanocrystalline Magnetic Shields

**DOI:** 10.3390/ma18020330

**Published:** 2025-01-13

**Authors:** Shuai Kang, Wenfeng Fan, Jixi Lu, Wei Quan

**Affiliations:** 1 Hangzhou Institute of National Extremely-Weak Magnetic Field Infrastructure, Hangzhou 310028, China; 2 School of Instrumentation and Optoelectronic Engineering, Beihang University, Beijing 100191, China; 3 The Institute of Large-Scale Scientific Facility and Centre for Zero Magnetic Field Science, Beihang University, Hangzhou 310051, China; 4 Hefei National Laboratory, Hefei 230088, China

**Keywords:** nanocrystalline, magnetic noise, finite element method

## Abstract

Depending on high permeability, high Curie temperature, and low eddy current loss noise, nanocrystalline alloys, as the innermost layer, exhibit great potential in the construction of cylindrical magnetic shielding systems with a high shielding coefficient and low magnetic noise. This study compares a magnetic noise of 1 Hz, simulated by the finite element method (FEM), of a cylindrical nanocrystalline magnetic shield with different structural parameters based on the measured initial permeability of commercial Fe-based nanocrystalline (1K107). The simulated results demonstrate that the magnetic noise is irrelevant to the pump and probe hole diameter. The magnetic noise of a nanocrystalline cylinder with a fixed length gradually increases with the rise in aspect ratio. The radial and axial magnetic noise of a nanocrystalline cylinder with a fixed diameter can reach optimal values when the aspect ratio is 1.3 and 1.4, respectively. The layer thickness of a nanocrystalline cylinder is negatively correlated to magnetic noise. Additionally, by comparing the 1 Hz magnetic noise of a cylindrical nanocrystalline magnetic shield with varying initial permeability, it can be concluded that an increase in loss factor results in an increase in magnetic noise. These results are useful for the design of a high-performance passive magnetic shield with low magnetic noise.

## 1. Introduction

Long-endurance and high-precision inertial navigation technology composed of a gyroscope and accelerometer is defined as an exclusively interference-free and fully autonomous navigation technology [1,2]. In recent years, atomic gyroscopes using Spin-Exchange Relaxation-Free (SERF) technology, which possesses a theoretical bias stability limit of 10^−8^°/h, have become a research hotspot in the field of inertial navigation because of the rapid development of quantum precision measurement technology [1,3]. Nevertheless, the accuracy of SERF inertial measurement instruments is still far below theoretical precision, and one of the reasons restricting the improvement of the precision is magnetic noise error [4].

The high-performance passive magnetic shielding system can effectively shield external magnetic fields, thereby providing a near-zero field environment for the normal operation of SERF inertial measurement instruments [5,6]. Due to their high permeability, resulting in a shielding coefficient of up to 10^6^, permalloy materials have been widely used to manufacture high-performance passive magnetic shields [7,8,9]. Unfortunately, the high shielding factor can bring a weak residual magnetic environment, but its low resistivity often results in significant Johnson current noise, thereby limiting the improvement of the accuracy of inertial measurement instruments. The bias error-induced magnetic noise will cause inertial measurement instruments to produce bias drift, leading to increased navigation inaccuracy [7,10,11,12,13]. MnZn ferrite materials with a higher electrical resistivity (<1 Ω m) than permalloy and that remarkably reduce magnetic noise have been increasingly used as the innermost layer of magnetic shielding systems [13,14,15,16,17]. As a result, high-performance passive magnetic shields consisting of permalloy as the outer layer and MnZn ferrite as the inner layer can achieve a low residual magnetic shield and magnetic noise [10,18]. Nevertheless, the Curie temperature of MnZn ferrite is low, and there are other problems, such as the unstable magnetic properties at high temperatures, high coercivity, and large remanence and gradient after demagnetization [19]. In addition, manufacturing a large-sized MnZn ferrite is difficult and expensive [7,19,20]. With the aim of improving the performance of SERF inertial measurement instruments and reducing the volume and cost, there is an urgent need to develop new magnetic shielding materials.

Nanocrystalline alloys, a new type of soft magnetic material, have advantages such as high saturation magnetization, low coercivity, high permeability, high Curie temperature, and low loss [21,22]. Moreover, the eddy current loss can be significantly suppressed due to the distinctive nanostructure and reduced thickness [23,24]. Therefore, they have achieved extensive applications in low-frequency fields such as power, industrial power, rail transit, and digital electronics [25]. Among numerous nanocrystalline alloys, Fe-based nanocrystalline alloys harvest an initial permeability of 10^4^–10^6^ and high-temperature stability, thereby demonstrating the potential to replace MnZn ferrites and be applied in low-frequency magnetic shields for SERF inertial measurement instruments. For instance, Liu et al. [19] compared the magnetic noise characteristics of the innermost amorphous alloy and nanocrystalline alloys of a magnetic shielding system and proved that the nanocrystalline magnetic shield achieved lower magnetic noise. Fu et al. [10] demonstrated that the nanocrystalline magnetic shield, as the innermost layer, generated the least magnetic noise compared to permalloy and MnZn ferrite. However, there are few studies on the magnetic noise of cylindrical nanocrystalline magnetic shields with different size parameters and initial permeability.

SERF inertial measurement instruments are mainly sensitive to low-frequency signals because of a narrow bandwidth. Herein, the 1 Hz magnetic noise of an Fe-based nanocrystalline magnetic shield with different structural parameters, including the pump and probe hole diameter, the aspect ratio, and the layer thickness, was simulated by using the commercial software ANASYS Electronic Desktop, 2021. The simulated 1 Hz magnetic noise of a nanocrystalline magnetic shield with an initial permeability range of about 40,000–180,000 was compared, and the relationship between the magnetic noise and the loss factor was established. These simulated results can offer guidance for the selection of nanocrystalline materials and the design of cylindrical nanocrystalline magnetic shields.

## 2. Method

### 2.1. Measurement Method of Relative Complex Permeability

**Figure 1:**
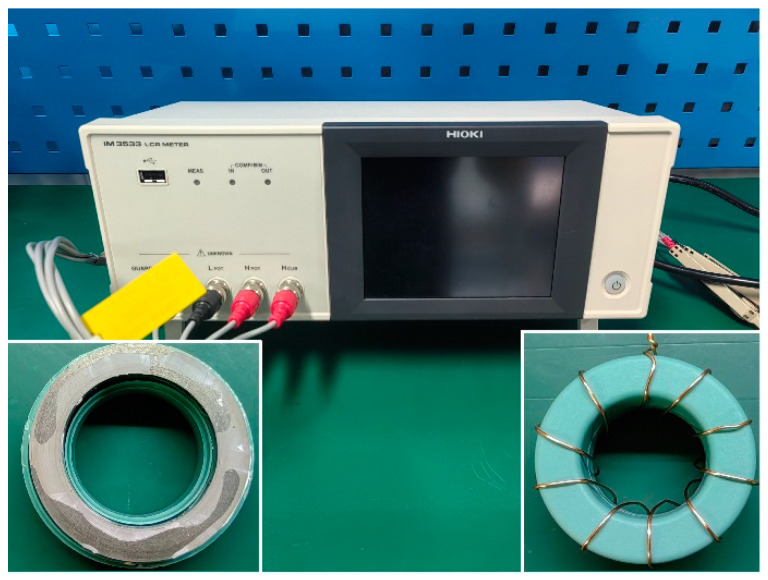
The IM 3533 LCR meter and Fe-based nanocrystalline magnetic ring.

To evaluate the magnetic noise of a cylindrical nanocrystalline magnetic shield, the relative complex permeability of nanocrystalline alloys must be measured based on the impedance measurement method. As shown in Figure 1, a coil with 10 turns was wound over the circular nanocrystalline magnetic ring with dimensions of 30 mm × 20 mm × 10 mm (outer diameter × inner diameter × height). The LCR meter was used to measure the inductance (*L*) and resistance (*R*). The real part (*μ*′) and imaginary part (*μ*″) of relative complex permeability were calculated by using the following equation [26]:

(1) $$\mu^{\prime }=\frac{l_{e}}{\mu_{0}A_{e}N_{coil}^{2}}L$$

(2) $$\mu^{\prime \prime }=\frac{l_{e}}{2\pi f\mu_{0}A_{e}N_{coil}^{2}}\left(R-R_{w}\right)$$

where *l_e_* and *A_e_* represent the equivalent magnetic path length and the cross-sectional area of the nanocrystalline magnetic ring, respectively; *f* is the excitation frequency; *μ*_0_ is the permeability of vacuum; *N_coil_* is the number of turns; *L* and *R* are the measured inductance and resistance, respectively; and *R_w_* is the resistance of the wire. The relationship between *R* and *R_w_* is as follows:

(3) $$R=A\cdot f+R_{w}$$

where *A* is a constant. By measuring *R* under the different excitation frequencies (20–110 Hz), *R_w_* was acquired after the linear fitting. Because the LCR meter cannot directly apply the specific magnetic field strength to the magnetic ring, the magnetic field intensity needs to be converted into current according to the following formula:

(4) $$I=\frac{Hl_{e}}{N_{coil}}$$

where *I* is the applied current, and *H* is the corresponding magnetic field strength.

In a multilayer magnetic shielding cylinder, the innermost nanocrystalline layer experiences a weak low-frequency magnetic environment. The relative complex permeability of nanocrystalline materials at near-zero magnetic fields can be obtained by using the Rayleigh relationship, which is expressed by [7]

(5) $$\mu_{a}=\mu_{i}+\eta H$$

in which *μ_a_* is the amplitude permeability; *μ_i_* represents the initial permeability; and *η* is the Rayleigh constant.

### 2.2. Method of Simulation

#### 2.2.1. Structure of Cylindrical Nanocrystalline Magnetic Shield

**Figure 2:**
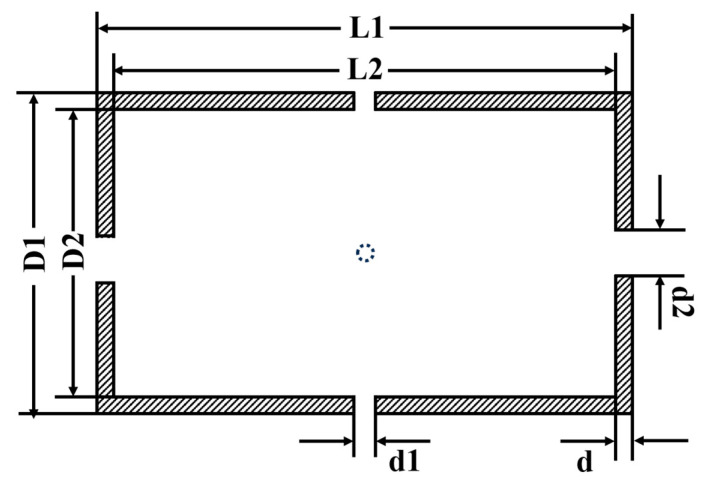
The structure of the cylindrical nanocrystalline magnetic shield.

The structure of the cylindrical nanocrystalline magnetic shield is presented in Figure 2. The pump and probe laser are passed through the axial and radial holes, respectively. L1 and L2 are the outer and inner lengths, respectively. D1 and D2 represent the outer and inner diameters, respectively. d1 and d2 are the probe and pump hole diameters, respectively. t is the layer thickness.

#### 2.2.2. Simulation Method of Magnetic Noise

**Figure 3:**
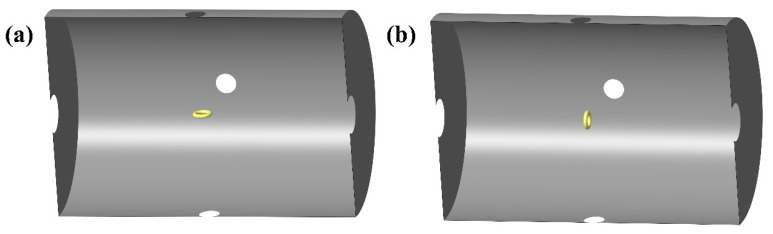
Simulation model of magnetic noise of cylindrical nanocrystalline magnetic shield at orthogonal directions: (**a**) radial direction and (**b**) axial direction.

The magnetic noise of nanocrystalline at a specific location in the magnetic shield can be calculated from the power loss generated by an excitation coil located at the same point based on the generalized Nyquist relation [7,14,27].

(6) $$\delta B_{magn}=\sqrt{\frac{4k_{B}T\mu^{\prime \prime }}{2\pi f}}\frac{\sqrt{\int_{V}H^{2}dV}}{A_{hc}I}$$

in which *k_B_* is the Boltzmann constant; *T* is the temperature (K); *μ*″ represents the imaginary part of relative complex permeability; *f* is the frequency of the excitation coil; *H* is the amplitude of the magnetic field intensity generated by a current (*I*) flowing in the excitation coil; and *A_hc_* represents the sectional area of the excitation coil. As revealed in Figure 3, an excitation coil with a current of 0.1 A and an outer diameter of 1 mm was placed at the center of the cylindrical nanocrystalline magnetic shield. Figure 3a demonstrates the simulation model of the radial magnetic noise, in which the excitation coil was placed along the radial direction of the magnetic shield. It can be observed from Figure 3b that the excitation coil in the simulation model of the axial magnetic noise was placed along the axial direction of the magnetic shield. The LCR meter was used to acquire *μ*″. *∫_V_H*^2^*dV* was calculated by using the commercial finite element software (ANSYS Electronic Desktop). Because the sensor in SERF inertial measurement instruments is located at the center of the magnetic shielding cylinder, only the magnetic noise at the center of the cylindrical magnetic shield was simulated in this study. According to the above formula, the magnetic noise was proportional to the temperature. However, the permeability of materials is closely related to the temperature. With the increase in temperature, the saturation flux density of nanocrystalline materials diminishes, and the gradually weakened coupling effect between the grains of nanocrystalline materials results in an increase in average magnetocrystalline anisotropy, thereby leading to a decrease in permeability. In addition, decreased magnetocrystalline anisotropy caused by external mechanical stress results in a decline in permeability. Therefore, the change in magnetic noise is influenced by the comprehensive results of the temperature, mechanical stress, and permeability.

## 3. Measurement and Simulation Results

**Figure 4:**
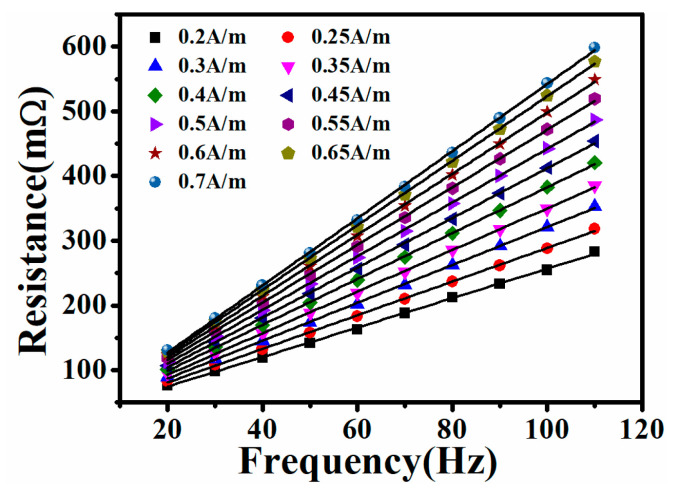
The measured resistance (*R*) throughout a magnetic field intensity of 0.2–0.7 A/m at an excitation frequency of 20–110 Hz.

**Figure 5:**
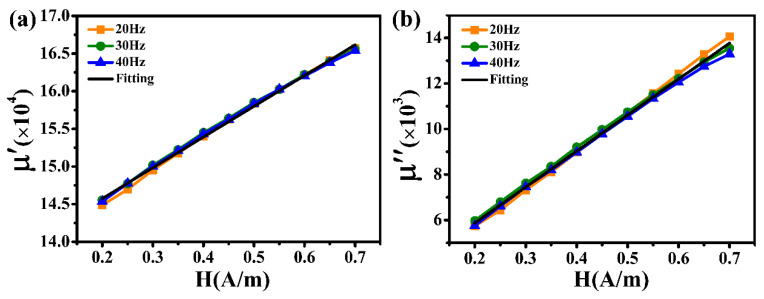
(**a**) The real part (*μ*′) and (**b**) the imaginary part (*μ*″) of the relative complex permeability of Fe-based nanocrystalline (NC-1) with a magnetic field intensity of 0.2–0.7 A/m, with excitation frequencies of 20 Hz, 30 Hz, and 40 Hz.

The magnetic noise of a cylindrical magnetic shield is usually determined by the innermost part. As the innermost magnetic shield, the nanocrystalline material is in a near-zero magnetic field environment. Consequently, based on the above theoretical analysis, the simulation calculation of the magnetic noise is closely related to the real part (*μ*′) and the imaginary part (*μ*″) of the relative complex permeability at zero magnetic field strength. Firstly, a current (*I* = 1.57 mA) corresponding to a magnetic field intensity of 0.2 A/m was applied to the excitation coil by an IM 3533 LCR meter. The resistance (*R*) and inductance (*L*) were acquired by changing the excitation frequencies of 20 Hz, 30 Hz, and 40 Hz. The current was then increased following a fixed gradient. *R* and *L* were measured under different magnetic field intensities. Based on the aforementioned Equations (1) and (2), the values of *μ*′ and *μ*″ for the relative complex permeability may be computed throughout the magnetic field intensity range of 0.2 A/m to 0.7. Considering the Rayleigh region under the weak magnetic field, the initial permeability at zero magnetic fields can be obtained after the liner fitting. The measured *R* results throughout a magnetic field intensity of 0.2–0.7 A/m at an excitation frequency of 20–110 Hz are displayed in Figure 4. There is a good linear relationship between *R* and *f* under the same magnetic field strength. After the linear fitting, the wire resistance (*R_w_*) throughout a magnetic field intensity range of 0.2–0.7 A/m is recorded as 28.93 mΩ, 28.68 mΩ, 28.35 mΩ, 28.33 mΩ, 28.06 mΩ, 27.51 mΩ, 27.18 mΩ, 26.54 mΩ, 25.88 mΩ, 24.65 mΩ, and 23.43 mΩ, respectively. The difference in the above *R_w_* value may be ascribed to the measurement error. Figure 5 shows the measured values of *μ*′ and *μ*″ for the Fe-based nanocrystalline magnetic ring (NC-1, 1K107) obtained from Advanced Technology & Materials Co., Ltd., in a magnetic field intensity range of 0.2–0.7 A/m. It can be observed that there is no relationship between *μ*′ (or *μ*″) and the excitation frequency at low magnetic field strength. After the fitting, the values of *μ*′ and *μ*″ for NC-1 at a near-zero magnetic field are 137,580 and 2702, respectively. Unlike ferrite magnetic rings, nanocrystalline magnetic rings are made by winding thin ribbons in a multilayer, which increases the gaps between layers (Figure 1: inset in the bottom left corner). Therefore, the initial *μ*′ and *μ*″ values are closely related to the lamination factor defined as the ratio of effective thickness to the total thickness of the magnetic ring. There are studies pointing out that the relationship between the permeability and the lamination factor can be expressed as the following equation [28,29]:

(7) $$\mu_{m}=F\mu_{s}+\left(1-F\right)\mu_{0}$$

where *μ_m_* represents the permeability of the magnetic ring; *F* is the lamination factor; *μ_s_* is the permeability of a single ribbon; and *μ*_0_ is the permeability of free space. It is worth noting that *μ_s_* is far higher than *μ*_0_, so *μ_m_* is roughly equivalent to the value of *Fμ_s_*. According to Equation (7), the values of *μ*′*_s_* and *μ*″*_s_* for NC-1 at a near-zero magnetic environment are 176,385 and 3464, respectively.

**Figure 6:**
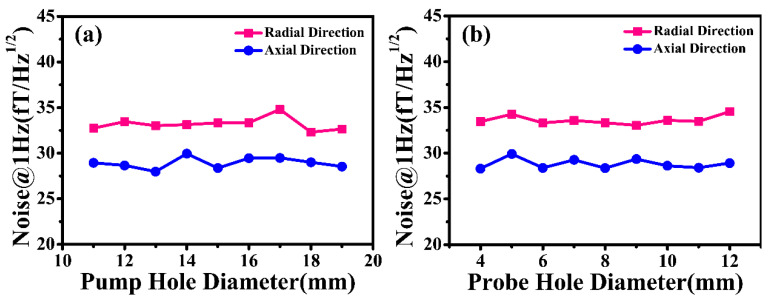
The simulated 1 Hz magnetic noise of the cylindrical nanocrystalline magnetic shield considering (**a**) different pump hole diameters and (**b**) different probe hole diameters.

Table 1 presents the initial size parameters, including the inner length and diameter, the pump and probe hole diameter, and the layer thickness of the nanocrystalline cylinder. On this basis, the 1 Hz magnetic noise of a cylindrical nanocrystalline (NC-1) magnetic shield with different size parameters was simulated by commercial ANSYS software (2021), which guided us in designing the high-performance passive magnetic shielding system. Previous studies have demonstrated that hysteresis loss noise is dominant in the overall magnetic noise at low frequencies (<100 Hz) in nanocrystalline magnetic shields [23]. Therefore, only hysteresis loss noise at a frequency of 1 Hz is analyzed in this article. Figure 6 demonstrates the correlation between magnetic noise at a 1 Hz frequency, a pump hole diameter of 11–19 mm, and a probe hole diameter of 4–12 mm. It can be clearly seen from Figure 6 that the radial and axial magnetic noise of the NC-1 magnetic shield is independent of the size of the pump and probe hole diameter. Another phenomenon where the radial magnetic noise is greater than the axial one is observed as well. The details are as follows: the radial magnetic noise of the NC-1 magnetic shield is approximately 33.32 fT/Hz^1/2^, while an axial magnetic noise of about 28.36 fT/Hz^1/2^ is acquired.

**Figure 7:**
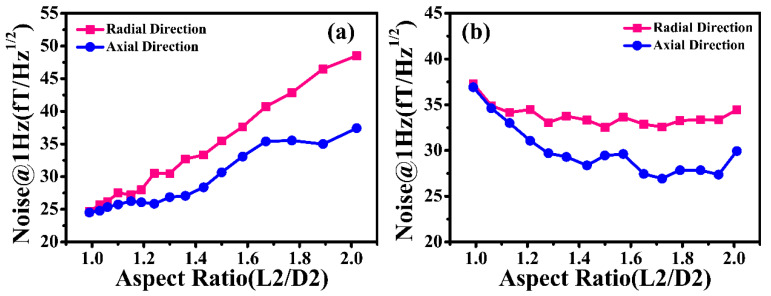
The simulated 1 Hz magnetic noise of the cylindrical nanocrystalline magnetic shield considering an aspect ratio range of 1–2: (**a**) the different diameter and (**b**) the different length.

The influence of the aspect ratio (L2/D2) on a magnetic noise of 1 Hz was also simulated, as was the pump and probe hole diameter. As revealed in Figure 7a, the magnetic noise of the NC-1 magnetic shield increases with the increase in the aspect ratio when the length remains constant. When the aspect ratio is 1, the radial and axial magnetic noise are 24.66 fT/Hz^1/2^ and 24.53 fT/Hz^1/2^, respectively. As the aspect ratio increases to 2, these figures climb to 48.51 fT/Hz^1/2^ and 37.42 fT/Hz^1/2^, respectively. At the same time, it can be clearly observed that the difference between them gradually decreases when the aspect ratio decreases. Figure 7b illustrates the magnetic noise of the NC-1 magnetic shield with a fixed diameter under the different aspect ratios. The magnetic noise first decreases and then remains basically unchanged when the aspect ratio increases from 1 to 2. Specifically, the radial magnetic noise shows a downward trend when the aspect ratio is below 1.3. The radial magnetic noise basically stays constant once the aspect ratio is greater than 1.3. For the axial magnetic noise, the optimal aspect ratio is about 1.4. Because of the restriction in volume, the correct diameter and length of the cylindrical nanocrystalline magnetic shield should be employed in the design process of the high-performance passive magnetic shielding system.

**Figure 8:**
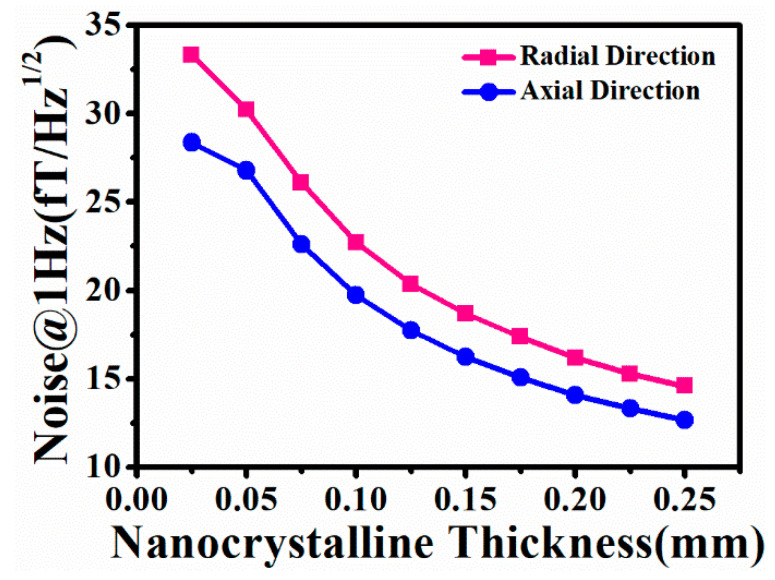
The simulated 1 Hz magnetic noise of the cylindrical nanocrystalline magnetic shield considering the different layer thicknesses.

The layer thickness of the nanocrystalline magnetic shield is another factor that affects the magnetic noise. As a result, it is necessary to analyze the 1 Hz magnetic noise of a nanocrystalline magnetic shield with different layer thicknesses. Figure 8 illustrates the relationship between the 1 Hz magnetic noise and the layer thickness. It can be clearly seen that increasing the layer thickness can lead to a decrease in the magnetic noise. The details are as follows: the radial and axial magnetic noise of the single-layer nanocrystalline magnetic shield are 33.32 fT/Hz^1/2^ and 28.36 fT/Hz^1/2^, respectively. When the number of nanocrystalline layers increases to ten, the magnetic noise can decrease to 14.59 fT/Hz^1/2^ and 12.67 fT/Hz^1/2^, respectively. Although the magnetic noise can be reduced by increasing the layer thickness of the nanocrystalline magnetic shield, the layer thickness that is too excessive is not suitable for the winding of a cylindrical nanocrystalline magnetic shield. Consequently, the correct layer thickness is crucial during the design process of a nanocrystalline magnetic shielding cylinder.

**Figure 9:**
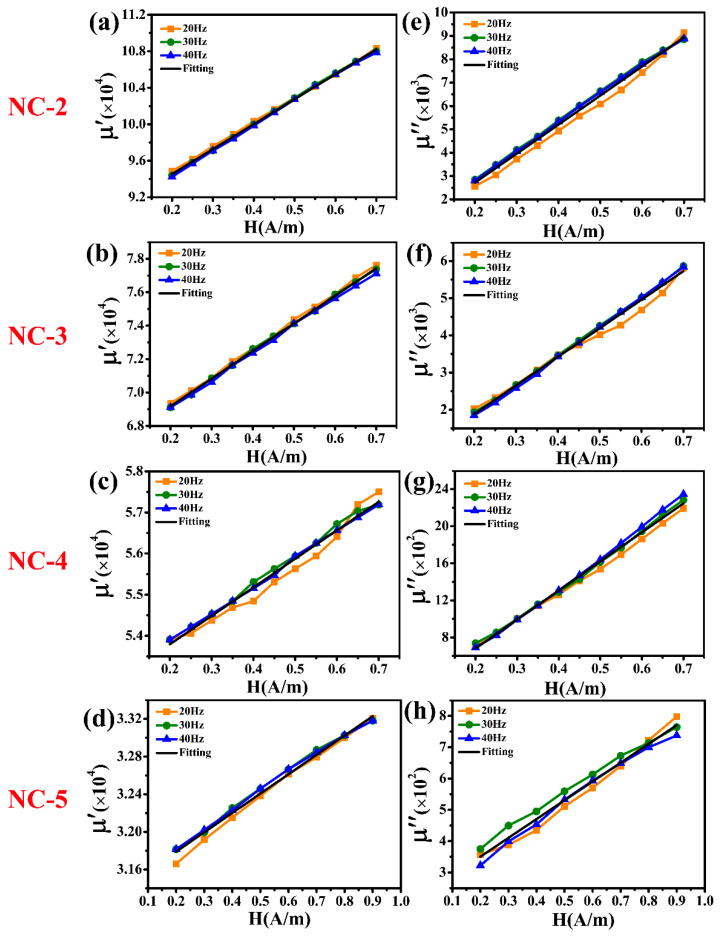
(**a**–**d**) The real part (*μ*′) and (**e**–**h**) the imaginary part (*μ*″) of the relative complex permeability of the different Fe-based nanocrystalline magnetic rings at an excitation frequency of 20 Hz, 30 Hz, and 40 Hz.

With the aim of evaluating the 1 Hz magnetic noise level of the commercial nanocrystalline magnetic shield, several Fe-based nanocrystalline magnetic rings, each with a different initial permeability, were chosen. Figure 9 presents the measured values of *μ*′ and *μ*″ of the other four commercial Fe-based nanocrystalline magnetic rings (NC-2, NC-3, NC-4, and NC-5, 1K107). At low magnetic field intensity, their *μ*′ and *μ*″ exhibit a good linear relationship with the increase in magnetic field strength, similar to those of NC-1. As expected, *μ*′ and *μ*″ are irrelevant to the excitation frequency. After the linear fitting, the initial *μ*′ values of NC-2, NC-3, NC-4, and NC-5 are 89,085, 65,877, 52,411, and 31,378, respectively, and their initial *μ*″ values are 222, 346, 55 and 230, respectively. Table 2 presents the lamination factor and initial permeability of all nanocrystalline materials. The lamination factor (*F*) of the NC-1, NC-2, and NC-3 magnetic rings from Advanced Technology & Materials Co., Ltd. (Beijing, China) is 0.78, while that of NC-4 and NC-5 magnetic rings from Zhejiang Crystal Core Magnetic Industry Co., Ltd. (Jiaxing, China) is 0.8. Therefore, the initial permeability range of single-layer nanocrystalline materials is 40,000–180,000 after the theoretical calculation based on Equation (7).

The 1 Hz magnetic noise of all nanocrystalline magnetic shields is compared on the basis of the above-calculated results of *μʹ_s_* and *μ″_s_*. Table 3 presents the simulated results of the radial and axial magnetic noise at a frequency of 1 Hz. NC-1 exhibits the largest initial permeability, but 1 Hz magnetic noise is not the lowest frequency registered. NC-5, achieving the smallest initial permeability, presents the highest magnetic noise (1 Hz), including a radial magnetic noise of 43.65 fT/Hz^1/2^ and an axial magnetic noise of 37.73 fT/Hz^1/2^. The NC-4 magnetic shield presents the lowest magnetic noise, with a radial magnetic noise of 13.00 fT/Hz^1/2^ and an axial magnetic noise of 11.34 fT/Hz^1/2^. In order to screen the nanocrystalline materials in the early design stage of the high-performance passive magnetic shield, the loss factor (*α* = *μ*″*μ*_0_/*μ*′^2^) was introduced in this study. As revealed in Table 3, the larger the value of *α*, the greater the magnetic noise. Consequently, the nanocrystalline material can be selected according to the value of *α*.

## 4. Conclusions

This paper presents a simulation of a 1 Hz magnetic noise for a cylindrical nanocrystalline magnetic shield, utilizing the measured results of *μ*′ and *μ*″ of Fe-based nanocrystalline materials. The simulation, conducted using the finite element method (FEM), explored various structural parameters, including the pump and probe hole diameter, aspect ratio, and layer thickness. The simulated results demonstrate that the magnetic noise is independent of the pump and probe hole diameter. When increasing the aspect ratio from 1 to 2, the magnetic noise of the nanocrystalline cylinder with the fixed length gradually increases. For the nanocrystalline cylinder with the fixed diameter, the magnetic noise first declines and then basically stays constant. The optimal aspect ratio is about 1.3 for the radial magnetic noise and approximately 1.4 for the axial one. The magnetic noise of cylindrical nanocrystalline magnetic shields with different layer thicknesses exhibits a downward trend with increasing layer thickness. Additionally, the 1 Hz magnetic noise of the nanocrystalline cylinder with an initial permeability range of approximately 40,000–180,000 was simulated. The cylindrical nanocrystalline magnetic shield, which has the smallest loss factor, achieves the lowest magnetic noise. When initial *μ*′ and *μ*″ values are 65,514 and 69, respectively, the minimal radial and axial magnetic noise recorded are 13.00 fT/Hz^1/2^ and 11.34 fT/Hz^1/2^. The above work can be used to guide the design of the high-performance nanocrystalline passive magnetic shield.

## Figures and Tables

**Table 1:** Size parameters of the cylindrical nanocrystalline magnetic shield.

Parameter Name	Parameter Value
Inner length	117 mm
Inner diameter	82 mm
Layer thickness	0.025 mm
Pump hole diameter	15 mm
Probe hole diameter	8 mm

**Table 2:** Lamination factor and initial permeability of Fe-based nanocrystalline at a near-zero magnetic field.

Sample	Lamination Factor (F)	*μ*′	*μ*″	*μ*′_*s*_	*μ*″_*s*_
NC-1	0.78	137,580	2702	176,385	3464
NC-2		89,085	222	114,212	285
NC-3		65,877	346	84,458	444
NC-4	0.8	52,411	55	65,514	69
NC-5		31,378	230	39,223	288

**Table 3:** Magnetic noise at a frequency of 1 Hz and loss factor of all nanocrystalline magnetic shields.

Sample	Radial Magnetic Noise (fT/Hz^1/2^)	Axial Magnetic Noise (fT/Hz^1/2^)	Loss Factor *α*
NC-1	33.32	28.36	1.113 × 10^−7^
NC-2	14.69	12.45	2.185 × 10^−8^
NC-3	24.71	20.87	6.224 × 10^−8^
NC-4	13.00	11.34	1.608 × 10^−8^
NC-5	43.65	37.73	1.872 × 10^−7^

## Data Availability

The original contributions presented in this study are included in the article. Further inquiries can be directed to the corresponding author.
